# The use of probiotic *L. fermentum* ME-3 containing Reg’Activ Cholesterol supplement for 4 weeks has a positive influence on blood lipoprotein profiles and inflammatory cytokines: an open-label preliminary study

**DOI:** 10.1186/s12937-016-0213-6

**Published:** 2016-10-28

**Authors:** Tiiu Kullisaar, Kersti Zilmer, Tiit Salum, Aune Rehema, Mihkel Zilmer

**Affiliations:** Department of Biochemistry, Institute of Biomedicine and Translational Medicine, The Centre of Excellence for Genomics and Translational Medicine, Faculty of Medicine, University of Tartu, Ravila 19, Tartu, 50411 Estonia

**Keywords:** Blood serum lipid profile, *L. fermentum* ME-3, Oxidized LDL, HbA1c%, Human clinical trial

## Abstract

**Background:**

Cardiovascular diseases continue to be a challenge and burden to heath. The incidence of type 2 diabetes is increasing. Modifying the (common) risk factors of them is the key of longterm success. The aim of the study was to establish if the special composition of innovative food supplement Reg’Activ Cholesterol (RAC) has a positive influence to the human body cardiovascular-inflammatory and diabetic parameters.

**Methods:**

Forty-five clinically asymptomatic participants consumed an RAC containing an antioxidative and antiatherogenic probiotic *Lactobacillus fermentum* ME-3 (LFME-3) for 4 weeks. The parameters measured were total cholesterol, HDL cholesterol, LDL cholesterol, triglyceride, oxLDL, hsCRP, IL-6 and glycosylated haemoglobin (HbA1c%).

**Results:**

The cardiovascular and diabetes risk profile of the participants improved significantly after 4 weeks of the intervention. The reduction of total cholesterol (from 6.5 ± 1.0 to 5.7 ± 0.9 mmol/l, *p* = 9.90806E-11) was on the account of LDL cholesterol as the HDL cholesterol level rose from 1.60 ± 0.31to 1.67 ± 0.34mml/l, *p* = 0.01. HbA1c% was reduced from 5.85 ± 0.28 to 5.66 ± 0.25 *p* = 4.64E-05 and oxLDL decreased from 84 ± 20 to 71 ± 15 U/l, *p* = 4.66292E-08.

**Conclusions:**

The consumption of RAC in clinically asymptomatic volunteers with borderline-high values of risk factors for cardiovascular disease (BMI, HbA1c%, LDL cholesterol) for 4 weeks had a positive effect on blood lipoprotein, oxidative stress and inflammatory profile. There are no human trials published before with RAC.

**Trial registration:**

The trial described here isa n open label pilot study within the framework of a larger special clinical trial (ISRCTN55339917) [Accessed 20 Feb 2016].

## Background

Cardiovascular disease (CVD) performs the number one cause of death worldwide and continues to be a major economic and health burden globally [1]. Atherosclerosis is the pathophysiological process underlying CVD. The signals starting the diseases are not ultimately understood and a role of an unbalanced intestinal microbiota in atherosclerosis pathogenesis is discussed [2]. Among the risk factors of CVD hypercholesterolemia and an elevated level of blood triglycerides (TG) are generally accepted. An elevated level of low-density lipoprotein (LDL) is a major measurable risk factor for CVD thus blood lipid lowering strategy has been a primary target for both in primary and secondary cardiovascular disease prevention to date [3]. The role of an elevated level of oxidized LDL (oxLDL) in the pathogenesis of atherosclerosis is also stressed [4] thus all mechanisms leading to extensive oxidation of the LDL particles are unfavorable from the point of view of CVD.

Inflammation and non-specific haemoglobin (Hb) glycation are important pathogenetic factors of CVD and diabetes. Abnormal Hb glycation (an elevated HbA1c%) is linked to the pathophysiology of type 2 diabetes mellitus (T2DM) effect on CVD. A permanent low-grade (or high-normal) inflammation (LGI) and an advanced glycation both can enhance the development of CVD as demonstrated in the ATTICA study that supported a positive association between LGI and diabetes [5]. LGI occurs typically in the vasculature and adipose tissue of a subject and is chronic in its nature. The parameter universally accepted to measure LGI is hsCRP [6]. It has been shown that triglyceride/HDL-cholesterol ratio can be used as a marker of insulin resistance (a prediabetes/ diabetes related marker) [7].

Although the study presented here is the first with RegActive Cholesterol formula, the probiotic strain L.*fermentum* ME-3 has been extensively tested both in humans and animals. *Lactobacillus fermentum* ME-3 (DSM 14241) (LFME-3) is a strain of human origin isolated from a healthy child [8]. The strain has both antimicrobial and antioxidative functional properties including the complete glutathione system [9–11]. Several benefits for health of fermented products containing LFME-3 have been demonstrated in various clinical studies including a favorable impact on postprandial lipemia and oxidative stress status [12–15]. Reg’Activ Cholesterol™ (VF Bioscience) has been formulated for promoting cardiovascular health. This innovative formulation combines LFME3 with others functional ingredients that are listed and described in the Materials section.

The aim of the study was to establish if a special formulation of Reg’Activ Cholesterol (RAC) comprising also LFME3 has a positive influence to the human body cardiovascular, inflammatory and diabetic parameters LDL cholesterol, TG, oxLDL, hsCRV, IL-6 and glycated haemoglobin (HbA1c%).

## Materials and methods

### Participants (subjects) and study design

Within the framework of a larger clinical trial (ISRCTN55339917) [16] forty five clinically healthy volunteers with borderline values for risk factors for cardioveascular diasease were recruited into a preliminary, open label study (Table 1).

**Table 1:** General data of 45 healthy volunteers in the open label study

Indices	Clinically healthy volunteers, *n* = 45
	An open label preliminary study
Sex (female/male)	35/10
Age	50–75
Body mass index (kg/m^2^)	26 ± 2.5

The needed sample size was calculated based on our previous experiments: if the difference in a parameter changes clinically significantly for at least 50 % of participants, it should show also a statistically significant difference between controls and the intervention/supplemented group. In our experiment the main parameters of interest (thus being the primary outcome measures) were the amount of LDL cholesterol, oxLDL and HbA1c %.

In the recruitment phase (open public call to participate) the inclusion criteria were age 50–75 years, BMI 24–30, clinically healthy (asymptomatic). The exclusion criteria were: a history of gastrointestinal disease, diabetes, food allergy, use of any antimicrobial agent or probiotics or an acute infection within the preceding 2 months; use of any regular concomitant medication including any non-steroidal anti-inflammatory drugs and antioxidant products or probiotics within at least the preceding 2 weeks; pregnancy or breastfeeding; any serious organ or systemic disease, eating disorder, extensive exercise, genetic hyperlipidemia, drug or alcohol abuse, smoking, active weight loss > 5 kg in prior 3 months; participation in other studies within the last 30 days/during the study and no wish to participate. Then the HbA_1c_ was measured (2 weeks before the actual intervention/test period started) and the participants with HbA_1c_ value between 5.7–6.2 (the value 5.7 being the lower end for prediabetes diagnostic criteria and although the upper value for prediabetes is 6.4 we considered 6.2 for our study to be more suitable to exclude possible undiagnosed diabetics) were included into the study. The participants were asked not to change their diet habits during the investigation period.

All participants signed their written informed consent and had the option of withdrawing from the study at any time. The Ethics Review Committee (ERC) on Human Research of the University of Tartu approved the study protocol. This study was carried out in accordance with the Declaration of Helsinki of the World Medical Association.

Each participant was evaluated for anthropometrical indices. Body mass index (BMI) was calculated as the weight (kg) divided by squared height (m^2^) [17]. The samples of fasting blood for the analysis set were collected two times: at the beginning and at the end of the trial period of administration of LFME3 containing capsules RAC for 4 weeks 2 capsules/day. The plasma samples were collected after an overnight fast and abstinence from any medications, tobacco, alcohol and tea or coffee. Samples were kept at −80 °C until analyzed.

RAC has been formulated for promoting cardiovascular health. This innovative formulation combines LFME3 with others functional ingredients that have been used in food supplements due to their antioxidative properties (Table 2).

**Table 2:** The composition of the Reg’Active Cholesterol Capsule

Ingredients	Quantity per recommended daily dose (2 capsules)	% of RDA
Red Yeast rice	666 mg (10 mg monacolin K)	-
*Lactobacillus fermentum* ME-3	60 mg (equals 6x 10^9^ LAB)	-
Ubiquinol (Kaneka QH^TM^)	30 mg	-
L-cysteine	30 mg	-
Vitamin E	10 mg	83 %
Vitamin B1	0.66 mg	60 %
Vitamin B6	1 mg	72 %
Vitamin B9	100 μg	50 %
Vitamin B12	1.5 μg	60 %

Other ingredients: maltodextrin (filling agent), magnesium salts of fatty acids (anti-caking agent), silica dioxide (anti-aggregating agent), vegetable capsule (hydroxyl-propyl-methyl-cellulose), soy lecithin

The strain LFME-3 is deposited in the Deutsche Sammlung von Mikroorganismen und Zellkulturen (German Collection of Microorganisms and Cell Cultures GmbH) under registration number DSM 14241. Molecular identification of the strain as LFME3 was confirmed by internally transcribed spacer polymerase chain reaction and 16SrRNA sequencing [18].

The markers for determination of the cardiovascular health were selected according to the suggestions by the NDA of European Food Safety Authority [19].

## Measurement of biochemical parameters

### Oxidized LDL

To measure oxidized LDL (oxLDL) the ELISA kit (Cat. No. 10-1143-01, Mercodia AB, Uppsala, Sweden) was used [20]. The solid phase two-site enzyme immunoassay is based on the direct sandwich technique in which two monoclonal antibodies are directed against separate antigenic determinants on the oxidized apolipoprotein B molecule. During incubation oxidized LDL in the sample reacts with anti-oxidized LDL antibodies bound to micro titration well. After washing a peroxidase conjugated anti-human apolipoprotein B antibody recognizes the oxLDL bound to the solid phase. After a second incubation and washing the bound conjugate was detected by reaction with 3,3’,5,5’-tetramethylbenzidine. The reaction is stopped by adding acid and the intensity of the color was measured spectrophotometrically at 450 nm and the results were presented as U/L.

Analyses of metabolic indices (plasma glucose and lipids: total cholesterol, LDL cholesterol, HDL cholesterol, IL-6, TG, hsCRP and HbA1c% were performed with standard laboratory methods using certified assays in the United Laboratories of the Tartu University Hospital, Estonia. Intervals for routine laboratory tests proposed by the Nordic Reference Interval Project [21] were used as references.

### Statistical analysis

Calculations were performed using commercially available statistical software packages (Statistics for Windows, Stat Soft Inc. and Graph Pad PRISM Version 2.0). All values are given as mean and standard deviation (mean ± SD). Statistically significant differences between the values before and after consumption of LFME-3 containing capsules were determined by using Student’s *t*-test. In all analyses, *p* values <0.05 were considered to be statistically significant.

## Results

The level of LDL cholesterol as well as total cholesterol and oxLDL decreased significantly in all participants and HDL cholesterol showed a tendency of improvement after 4 weeks of consumption of LFME3 containing food supplement RAC (Table 3). The level of inflammatory markers hsCRP and IL-6 as well as the level of HbA1c% decreased significantly after 4 weeks of administration of RAC. The use of RAC also declined the insulin resistance marker TG/HDL cholesterol ratio (Table 3).

**Table 3:** The cardiovascular markers and an insulin intolerance parameter (TG/HDLChlesterol) in 0 week and after 4 weeks consumption of probiotic LFME3 containing RAC capsules, 2 capsules/per day

Parameter	Asymptomatic subjects	
	*n* = 45	
	0 week	4 weeks
Cholesterol (mmol/l)	6.5 ± 1.0	5.7 ± 0.9
		*p* = 9.90806E-11
LDL cholesterol (mmol/l)	4.5 ± 0.9	3.7 ± 0.8
		*p* = 2.30351E-10
HDL cholesterol (mmol/l)	1.60 ± 0.31	1.67 ± 0.34
		*p* = 0.01
TG (mmol/l)	1.6 ± 0.4	1.4 ± 0.4
		*p* < 0.003
TG/HDL cholesterol	1.06 ± 0.43	0.88 ± 0.35
		*p* = 0.0002
hs CRP (mg/l)	2.9 ± 2.0	2.0 ± 1.0
		*p* = 0.007
HbA1c %	5.85 ± 0.28	5.66 ± 0.25
		*p* = 4.64E-05
oxLDL U/l	84 ± 20	71 ± 15
		*p* = 4.66292E-08
IL-6 (pg/ml)	2.2 ± 0.9	1.7 ± 0.5
		*p* < 0.00185

## Discussion

The limitations of our study design are mainly attributable to the lack of using a standardized diet prior and during the intervention. By using quite strict exclusion criteria we tried to minimize the influence of diet.

The elevated LDL cholesterol and blood triglyceride levels are well accepted risk factors of CVD [3]. Inflammation and elevation of HbA1c% are related to pathogenesis of both CVD and diabetes [5]. An elevated level of oxLDL has a role in the pathogenesis of atherosclerosis [4]. Thus additional good tools to control blood lipoprotein, oxidative stress and inflammatory profile are needed. This study investigated a special formulation of food supplement RAC and established positive effects on blood lipid profile, oxidized LDL level, inflammatory signature, HbA1c% and TG/HDL cholesterol ratio.

The creation of an innovative formulation RAC with different bioactive components was based on the previously obtained scientific information.

Gut microbiota forms an essential part of the complex ecosystem of the host that is involved in nutrition and health. A wide variety of host, microbiological, dietary and environmental factors affect the metabolic interrelations between gut mucosal epithelial cells and microbiota [22–25]. Organisms obtain a portion of cholesterol through nutrition and the major part is produced by highly-regulated biosynthesis in human body while dominating amount of cholesterol is eliminated from the organism as bile acids [26]. Among normal microbiota of gastrointestinal tract some species and strains of lactic acid bacteria are able to assimilate cholesterol from provided dietary products [27]. A meta-analysis of 13 probiotic studies indicates that a diet rich in probiotics decreases total cholesterol and LDL cholesterol concentration in plasma for participants with high, borderline high and normal cholesterol levels [28]. However, its relation to temporal colonization of digestive tract by probiotic strains and the duration of treatment remains to be evaluated.

Earlier studies have shown that the impact of probiotics on lipid metabolism markers of the host can be quite strain and host specific [29, 30]. The anti-oxidative and anti-atherogenic effect of LFME3 has been described previously in numerous in vitro and human trials with different dairy products such as goat milk, cheese and yoghurt [9, 12, 13, 15].

Insulin resistance is associated with cardiovascular disease pathogenesis. The evidence is piling up that he TG/HDL ratio can be used as a parameter to evaluate insulin resistance [7, 31–33]. After consumption of RAC capsules the TG/HDL cholesterol ratio decreased significantly.

Our previous data from 8 weeks versus data from 4 weeks showed that consumption of LFME3 enriched kefir, but not the placebo kefir, significantly decreased the level of LDL cholesterol [34]. Next, only the probiotic kefir in 8 weeks decreased significantly the ratio of TG/HDL cholesterol.

Thus, these data together show that compositions comprising LFME-3 help to prevent risk, alleviate the symptoms and treat metabolic syndrome related conditions (prediabetes, diabetes, cardiovascular disease).

Recently we found that using LFME3 causes a decline of blood level of myeloperoxidase (data not published) accepted as one of the main factors causing production of dysfunctional HDL particles [35].

Probably, there exist different mechanisms which lead to the positive effects of lactobacilli on various markers of lipid metabolism in human body [2, 36].

It is well known that during the passage of the upper part of intestinal tract probiotics have an ability to bind a certain amount of bile acids which leads to the loss of this amount from the enterohepatic recirculation. As a consequence, the liver elevates *de novo* production of new bile acids from cholesterol favoring a reduction of cholesterol levels. Some other possible mechanisms are as follows: LFME3 is characterized by a good activity of glycosyl-hydrolases, like alpha-galactosidase, beta-galactosidase, alpha-glycosidase, beta-glycosidase and beta-glycoronidase [37]. When LDL cholesterol levels are elevated a part of it is oxidized and subsequently taken up by the arterial wall to form atherosclerotic plaques. However, the cholesterol transport system based on HDL is responsible for facilitating the movement of cholesterol from tissues back to the liver.

The RAC contains monacolin K that is an inhibitor of cholesterol synthesis key-enzyme [38, 39]. The RAC formula also contains vitamin E and ubiquinol (vitamin Q), both working in the human body as powerful antioxidants. A study by Ceriello and colleagues also demonstrated the beneficial effects of targeting hyperglycemia and oxidative stress (OxS) and endothelial dysfunction simultaneously using insulin and an antioxidant (vitamin C) [40]. Targeting hyperglycemia and OxS simultaneously may help to ameliorate lipid abnormalities (e.g., elevated LDL levels) [41], and improve OxS which increases the susceptibility of LDLs to oxidation and glycation. This may prevent atherosclerosis and endothelial dysfunction. Food cysteine is the rate-limiting factor in cellular glutathione (GSH) biosynthesis. GSH, a well known cellular antioxidant and regulator of many body functions, is synthesized in the body from the amino acids L-cysteine, L-glutamic acid, and glycine. The sulfhydryl group of cysteine serves as a proton donor and is responsible for its biological activity. Thus, RAC contains L-cysteine and besides that LFME3 can transport and synthesise glutathione and has the ability of redox cycling of glutathione [11, 42]. Vitamins B1, B6, B9 and B12 have several widely known effects in human body, including improvement of overall energy workout, carbohydrate, lipid and amino acids (e.g. homocysteine) metabolism.

The capsule RAC has been specially formulated to improve cardiovascular health. The decline of IL-6 and hsCRP refer to an improvement of blood inflammatory status. However, it seems that such formula/composition might have an impact on cardiometabolic risk involving also prediabetes/diabetes. The triglyceride/HDL cholesterol ratio is a marker of insulin resistance (a prediabetes, diabetes related marker). We found that after consumption of RAC capsules the TG/HDL-Chol ratio decreased significantly. The data of our preliminary study show that there was also a decline in HbA1c%, a well accepted marker for prediabetes/diabetes. Of course the antidiabetic effect should be verified using longer randomized placebo controlled double blind trials.

Gastrointestinal OxS is associated with the non-specific glycation, which in turn may influence the long term level of blood sugar. Through the OxS and inflammation on the cellular level the up-regulation of pro-inflammatory cytokines may reduce glucose transporter type 4 (GLUT4) expression and translocation to the plasma membrane in human adipocytes and muscle cells, which may lead to the decreased insulin-stimulated glucose uptake [43, 44].

There are limitations of intensive treatment of hyperglycemia in preventing diabetic complications, because of adverse effects, which are linked to OxS. Maybe the simultaneous targeting of hyperglycemia and OxS could be more effective than intensive treatment of hyperglycemia in the management of T2DM [45]. A reduction in OxS and simultaneous cardiovascular risk factor control seems to be an ideal treatment strategy in T2DM patients [46]. Aside pharmaceutical and lifestyle correcting measures there is definitely room for food supplements and functional foods in the complex management of CVD and T2DM [4, 28].

## Conclusions

The consumption of RAC capsules in asymptomatic volunteers with borderline values of risk factors for cardiovascular disease (BMI, HbA1c%, LDL cholesterol) for 4 weeks had a positive effect on blood lipoprotein, oxidative stress and inflammatory profile.

## Abbreviations

CVD: Cardiovascular disease
Hb: Haemoglobin
HbA1c%: Glucosylated haemoglobin
HDL: High density lipoprotein
hsCRP: High sensitive C-reactive protein
LDL: Low density lipoprotein
LFME-3: *Lactobacillus fermentum* ME-3
LGI: Low grade inflammation
oxLDL: Oxidized low density lipoprotein
OxS: Oxidative stress
RAC: Reg’Activ Cholesterol
T2DM: Type 2 diabetes mellitus
TG: Triglycerides
